# Spontaneous idiopathic omental bleeding: a case report of rare cause of acute abdomen

**DOI:** 10.1093/jscr/rjad380

**Published:** 2023-06-30

**Authors:** Raju Sah, Dinesh Nalbo, Durga Neupane, Narendra Pandit

**Affiliations:** Department of Surgical Gastroenterology, Birat Medical College Teaching Hospital (BMCTH), Biratnagar, Nepal; Department of Surgical Gastroenterology, Birat Medical College Teaching Hospital (BMCTH), Biratnagar, Nepal; Department of Surgery, B.P. Koirala Institute of Health Sciences, Dharan, Nepal; Department of Surgical Gastroenterology, Birat Medical College Teaching Hospital (BMCTH), Biratnagar, Nepal

**Keywords:** acute abdomen, idiopathic omental hemorrhage, omentectomy, contrast CT, laparotomy, case report

## Abstract

Idiopathic omental hemorrhage is a rare cause of an acute abdomen, which is potentially life threatening. Here, we report a case of a 34-year-old male who presented to the emergency department with sudden, severe pain abdomen and abdominal distension for 1 day. There was no history of trauma, abdominal surgeries or any significant past medical history. The diagnosis was suspected on contrast computed tomography, which revealed hyperdense areas of blood in the peritoneal cavity with contrast extravasation from the omentum. The patient underwent successful emergency laparotomy, peritoneal lavage and greater omentectomy to achieve hemostasis.

## INTRODUCTION

Omental hemorrhage is a condition in which omental vessels rupture, causing bleeding in the peritoneal cavity [1]. It usually results from trauma, neoplasia, arterial aneurysm rupture, omental torsion, vasculitis or segmental arterial mediolysis [2, 3]. However, it can be idiopathic and spontaneous without any predisposing causes. Due to its rarity, and lack of its definite presentation history, it is very difficult to diagnose preoperatively. If diagnosed intraoperatively, it is best treated by excision of the bleeding omentum in toto due to its enormous vascularity [3].

Here, we present an interesting case of spontaneous omental bleed with hemoperitoneum, which was managed by surgery.

## CASE REPORT

A 34-year-old male presented to our emergency department, with a history of pain in periumbilical region and left iliac fossa for 1 day. Pain was sudden on onset, intermittent in nature and worsened on movement. It was associated with abdominal distention and multiple episodes of vomiting containing food particles. There was no history of trauma, abdominal surgery or any medical co-morbidities. On general physical examination, his blood pressure was 120/90 mm Hg and he had pulse rate of 110 beats per minute. He was pale. On abdominal examination, there was distension with peritonism. His blood investigation revealed drop in hemoglobin (Hb = 8.0 gm/dl). The ultrasound abdomen showed free fluid which on aspiration revealed blood.

**Figure 1:**
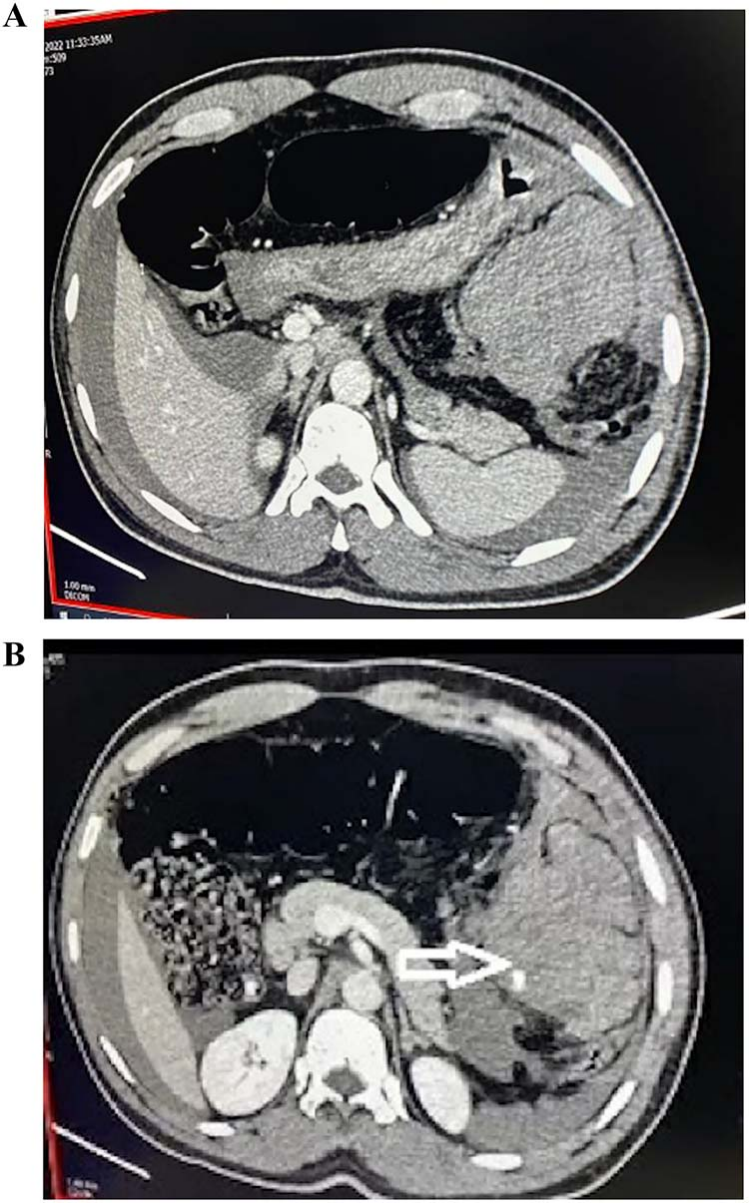
(**A**, **B**) CT abdomen and pelvis with IV contrast demonstrating hyperdense area of blood density suggestive of hematoma with eccentric focal intrinsic extravasation of contrast at left hypochondrium.

On further confirmation with contrast-enhanced computed tomography (CT) scan, it revealed hyperdense area of blood density, suggestive of hematoma, with eccentric focal intrinsic extravasation of contrast in the left hypochondrium—likely omental bleed (Fig. 1). Due to spontaneous hemoperitoneum, tachycardia and anemia—the patient was subjected to emergency laparotomy. At surgery, 2 l of intraperitoneal blood was suctioned. There was a large hematoma of size 10 by 12 cm noted in the left half of the greater omentum along the splenic flexure. Active ooze of blood from the greater omentum was noticed. Hence, greater omentectomy (partial) was done to control bleeding. No other solid organ injury or vessel aneurysm was identified.

On the fourth post-operative day, the patient developed complete wound dehiscence for which tension suture was applied. Subsequently, he improved and was discharged on Day 15.

The histopathological report of excised omentum showed thin lobules of adipose tissue consisting of mature adipocytes. Areas of congested vessels and hemorrhage were noted. There was no evidence of vasculitis or malignancy. At a follow-up of 6 months, he was free of symptoms.

## DISCUSSION

Isolated primary omental hemorrhage is a rare entity that is characterized only in case reports [3]. Secondary causes include trauma, neoplasm, aneurysm, vasculitis, adhesion or anticoagulant therapy [3]. It can occur to any age group patient without any predisposing illness or spontaneously, and it is usually observed more in men than in women.

Patient typically presents with sudden onset, severe abdominal pain with occasional nausea, vomiting or diarrhea [4–6]. Ultrasonography, CT scan (with aneurysm or contrast extravasation) and paracentesis are useful modalities to establish the diagnosis as was observed in the present case [5, 6]. Patient’s condition is often unstable and emergency operation is required for definitive diagnosis and treatment. On contrary, in our case, the patient was hemodynamically stable, but with signs of peritonism due to blood in the peritoneal cavity.

The treatment modality varies according to the availability of expertise and cause/site of aneurysm and bleeding. The bleeding can be managed by transcatheter arterial embolization, laparotomy or laparoscopy with omentectomy or simple ligation of bleeding artery [6–9].

In a case report by Ahmadi *et al*., a 53-year-old male presented with spontaneous omental hemorrhage, which was identified on the imaging performed for the diagnosis of the right iliac fossa pain, which required urgent laparotomy and omentectomy to achieve hemostasis [3]. Similarly, Kimura and Lyu *et al*. too reported a case of idiopathic omental hemorrhage in a 29-year-old male who presented with left upper quadrant pain, and after confirming the diagnosis on CT scan, an emergency laparotomy with partial omentectomy was done [10, 11].

The majority of reported cases proceeds to laparotomy and partial omentectomy; and omentectomy is preferred to ligation or transcatheter arterial embolization to rule out an underlying malignancy or aneurysm [6–9].

## CONCLUSION

Idiopathic omental bleeding is an infrequent condition where patient presents with acute abdomen in emergency. Diagnosis is established with contrast CT showing contrast extravasation. The entity is best managed with emergency laparotomy and omentectomy. With this case report, one should always have an index of suspicion for spontaneous omental bleed in the patient presenting to emergency with acute abdomen and hemoperitoneum so that timely intervention can be done to prevent further deterioration.
